# Engaging Adolescents and Young Adults in Decisions About Return of Genomic Research Results: a mixed-methods longitudinal clinical trial protocol

**DOI:** 10.21203/rs.3.rs-2819191/v1

**Published:** 2023-04-27

**Authors:** Amy Blumling, Michelle McGowan, Cynthia Prows, Kristin Childers-Buschle, Lisa Martin, John Lynch, Kevin Dufendach, Ellen Lipstein, Melinda Butsch Kovacic, Bill Brinkman, Melanie Myers

**Affiliations:** Cincinnati Children’s Hospital Medical Center; Mayo Clinic; Cincinnati Children’s Hospital Medical Center; Cincinnati Children’s Hospital Medical Center; Cincinnati Children’s Hospital Medical Center; University of Cincinnati; Cincinnati Children’s Hospital Medical Center; Cincinnati Children’s Hospital Medical Center; University of Cincinnati; Cincinnati Children’s Hospital Medical Center; Cincinnati Children’s Hospital Medical Center

## Abstract

**Background:**

To protect minors’ future autonomy, professional organizations have historically discouraged returning predictive adult-onset genetic test results and carrier status to children. Recent clinical guidance diverges from this norm, suggesting that when minors have genomic sequencing performed for clinical purposes, parents and children should have the opportunity to learn secondary findings, including for some adult-onset conditions. While parents can currently opt in or out of receiving their child’s secondary findings, the American Society of Human Genetics Workgroup on Pediatric Genetic and Genomic Testing suggests including adolescents in the decision-making process. However, it is not clear what factors young people consider when given the opportunity to learn genetic findings for themselves. We are examining adolescents’, young adults’, and parents’ (if applicable) decisions about learning genomic information for the adolescent.

**Methods:**

We are enrolling assenting (ages 13–17) adolescents and consenting (ages 18–21) young adults in a prospective genomic screening study to assess the choices they make about receiving individual genomic results. Participants use an online tool to indicate whether they want to learn their personal genetic risk for specific preventable, treatable, and adult-onset conditions, as well as carrier status for autosomal recessive conditions. We are examining 1) how choices differ between adolescent and young adult cohorts (as well as between adolescents/young adults and parents) and 2) decisional conflict and stability across study timepoints. Results are returned based on participants’ choices. Qualitative interviews with a subset of participants explore decisional stability, adolescent/young adult engagement with parents in decision-making, and the impact of learning pathogenic/likely pathogenic and carrier results.

**Discussion:**

This study explores decision making and decision stability between adolescents and parents (where applicable), as well as the ethical implications and impact of return of clinical-grade genetic research results to adolescents and young adults. The results of this study will contribute empirical evidence to support best practices and guidance on engaging young people in genetic research studies and clinical care that offer return of results.

## Background

To protect children’s future autonomy, professional organizations have historically recommended against predictive genetic testing of minors for conditions that would not affect medical management in childhood ((1, 2). Arguments in favor of protecting future autonomy typically claim there is a need to protect a child’s “open future” from decisions by parents that could foreclose the child’s future choices and undermine the child’s right to self-determination (3, 4). Recent scholarship has argued future autonomy is not an absolute bar against returning results, but is one interest among others, including the minor’s ability to express their interests and assent to learning adult-onset and carrier status results (5). Guidance from the American College of Medical Genetics and Genomics (ACMG) suggests that when minors have sequencing performed for clinical purposes, parents should have the opportunity to learn their child’s secondary findings, including for some adult-onset conditions (6, 7). These recommendations are based on the idea of best interest for both the child and the parent, as secondary findings (SFs) may be the only chance to identify a genetic risk factor in the parents (6, 7). The health of the parent is in the best interest of the child and returning secondary findings in a pediatric setting may be the only way to identify a pre-symptomatic parent with a variant as well.

Returning genetic risk information for adult-onset conditions in clinical pediatric settings remains controversial, particularly given adolescents’ limited legal and privacy rights to their personal genomic information (8). ACMG currently recommends sequencing an expert reviewed set of genes for opportunistic analysis when clinical sequencing is performed, regardless of the patient’s age, with the option for the parent of a pediatric patient to opt out of learning these secondary findings (7). However, the ACMG guidance is specific to sequencing performed based on clinical indication and does not apply to research settings. Opting in to learning secondary findings affords the parent the ability to learn more information on the child’s behalf. However, it places the parent at the center of decision-making, which may be incongruent with other professional society guidance that promotes increasingly involving adolescents and young adults in genetic decision-making as they mature (1).

Despite advocacy for engaging adolescents in this decision-making process for genetic testing (1, 7, 9, 10), best practices are unclear. There are increasing opportunities for minors to participate in predictive genomic screening research with parental permission (11, 12). Research with adolescents indicates that they want to be involved in decision-making about enrollment in genomic and biobank research studies and return of medically actionable findings (13–15). Parents and adolescents have suggested that involvement in the decision-making process about genomic research may depend on the adolescent’s age, maturity level, and personality (13, 16). However, parents and adolescents have varying perceptions as to the amount of decisional autonomy they would like each other to have when making decisions regarding learning genomic information (6, 7, 16–19). Additionally, studies have shown that adolescents and parents often differ in the amount and type of genomic screening information they desire to learn (17–19).

Our preliminary research suggests that adolescents often make different choices about learning genomic information than their parents. Furthermore, parents do not always agree with adolescents about who should be involved in the decision-making process (14, 18). In the Electronic Medical Records and Genomics Network Phase III (eMERGE III) study, 163 adolescent participants at Cincinnati Children’s Hospital Medical Center (CCHMC) and their parents made joint decisions to learn predictive genetic testing results about the adolescent. Overall, adolescents wanted to learn less than their parents, and adolescents had higher decisional conflict scores than their parents, suggesting higher rates of uncertainty in their choices (17, 19).

Building on these findings, we developed the “Engaging Adolescents in Decisions about Return of Genomic Results Study” to engage assenting adolescents (13–17 years) and consenting young adults (18–21 years) in decisions about learning personal genomic results and to evaluate the factors that influence their decisions, the stability of their decisions over time, and the impact of learning genomic results that reflect their choices. Our recruitment and enrollment approaches ensure a diverse study population that includes a wide age range of adolescents (assenting adolescents between 13–17 years of age who must involve a parent or legal guardian, as well as consenting young adults between 18–21 years of age who have the option of involving a parent or legal guardian). Further, we partner with community-engaged researchers to recruit participants with backgrounds that have been underrepresented in population genomic research.

## Methods

We are enrolling assenting adolescents’ (13–17 year olds) and consenting young adults’ (18–21 year olds) and a parent (optional for 18–21 year olds) in a National Human Genome Research Institute-funded clinical trial (ClinicalTrials.gov Identifier: NCT04481061) to utilize an electronic decision-making tool to learn genomic research results for preventable, treatable, and adult-onset conditions as well as carrier status for some autosomal recessive conditions.

### Development of Electronic Decision-Making Tool

During the first year of the project, we modified a paper-based decision tool and video used in eMERGE III (17). We used an iterative process to develop an online decision-making tool and videos to facilitate informed decision making about learning genomic information (20), with the assistance of community-based research participants and the CCHMC Biomedical Informatics team. Based on focus group feedback, we created a series of short videos and shared video modifications with focus group participants to evaluate modifications and solicit recommendations to further improve understandability and usefulness for a broader audience (20). While users can view all portions of the video on YouTube, short segments of the video are embedded within the final electronic version of the decision tool to provide just-in-time information about available choices. The electronic tool provides dynamic feedback to the user, indicating genetic conditions that would be included or excluded based on their choices regarding preventability, treatability, adult-onset, and carrier status. Participants can also select specific conditions for inclusion or exclusion. All responses are immediately captured and transmitted to REDCap (Research Electronic Data Capture), a secure, web-based software platform designed to support data capture for research studies (21, 22).

### Participants

Upon finalization of the educational videos and electronic decision tool, we began recruiting participants from communities within the greater Cincinnati area, pediatric hospital-affiliated clinics, and a pediatric hospital’s institutional biobank. We aim to enroll 440 adolescents and young adults, with a goal of no more than 65% of participants self-identifying as white. We established this goal after findings from our previous study suggested higher rates of parent-adolescent choice discordance among the Black/African American participants (17). However, in our previous study, less than 17% of participants self-identified as Black or African American; therefore, a larger sample proportion may help to generate more insight or generalizable results in the current study.

### Recruitment & Enrollment

Participants are recruited via IRB-approved materials and avenues, such as phone, letter, flyer, community health fairs, social media, snowball sampling, institutional research websites, community events, and trained community research advocates. Participants are recruited via IRB-approved materials and avenues, such as phone, letter, flyer, community health fairs, social media, snowball sampling, and institutional research websites, as well as community events, and trained community research advocates. Letters are also mailed to eligible families from the pediatric hospital’s institutional biobank who gave permission to be contacted for future research studies and families with an adolescent between the ages of 13–17 seen in a CCHMC primary care clinic since 2021. Envelopes are addressed to parents of adolescents aged 13–17 years, or directly to young adults aged 18–21 years who provided consent to participate in the biobank as an adult. If a participant transitions from a minor to consenting young adult during the study, they are reconsented into the study as an adult.

The research team has engaged several methods to promote participation among populations that have historically been underrepresented in genomic research. One example is our partnership with the We Engage 4 Health (WE4H) program (National Institute of General Medical Sciences (NIGMS) R25GM129808). The WE4H program is a community health project in the greater Cincinnati area that aims to improve health and science-related knowledge in the community. WE4H disseminates information through community research advocates-volunteers trained in communication and outreach to discuss health challenges and promote health and science within communities (23). The WE4H program study team utilizes short educational graphical stories at community centers and local community events such as health fairs and school functions to help inform the community of research opportunities.

### Inclusion & Exclusion Criteria

Inclusion criteria for the study include assenting adolescents ages 13–17 and a parent or legal guardian of the assenting adolescent. Consenting young adults ages 18–21 can choose whether to invite a parent to participate with them.

Exclusion criteria for the study include individuals with limited English proficiency, participants whose permanent address is greater than 100 miles from CCHMC, individuals with developmental disabilities that interfere with their ability to make decisions for themselves, and individuals who are regularly followed in clinic for a known or suspected genetic condition or have received a molecular diagnosis for a genetic condition. Full biological siblings of adolescent and young adult participants are not eligible to participate.

### Facilities & Performance Sites

Participants can select to participate in a virtual study visit via Zoom or an in-person study visit at CCHMC. Return of results occurs by mail, secure email, web conference, or phone. Completion of surveys occurs via mail, electronically, or over the phone. Interviews occur in-person at CCHMC, remotely via web conferencing, or over the phone.

### Data Collection

#### Specific Aim 1 – Genomic Testing Choices by Assenting Adolescents vs. Consenting Young Adults

Specific Aim 1 (SA1): To compare choices about learning genomic results, decisional conflict, and decision stability between assenting adolescents and consenting young adults. Participants use the digital decision tool developed for the study (https://demo.mygenechoices.com) to indicate preferences for learning personal genetic testing results for 75 genes related to 32 groups of conditions. Genes selected for analysis were informed by the ACMG (6, 24) recommendations for secondary analysis when sequencing is performed for clinical purposes and by the American College of Obstetricians and Gynecologists (ACOG) 2017 recommendations for carrier screening (25). A full list of genes and associated conditions is available in Supplement 1.

##### Procedures & Data Collection

**Figure 1:**
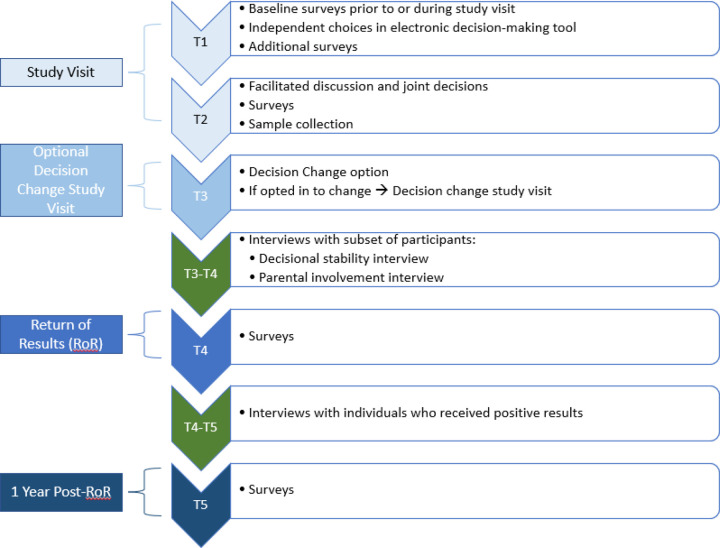
Study Timepoints

Methods and types of data collected throughout the study are listed below. Figure 1 and Table 1, listed after the Procedures & Data Collection section, provide a visual representation of the study visit and data collection timeline.

###### T1 – Independent Choices.

After providing informed consent, adolescent and parent participants are directed to six short videos about genetic testing, including pros and cons of testing and common thoughts and questions adolescents and young adults may have when deciding whether to learn genetic testing results about themselves (https://www.youtube.com/playlist?list=PLzQyg0rzF53S8S3fdyyko5Wr2ZKi2NdM-). Participants then complete baseline surveys prior to or at the beginning of the study visit. During the study visit adolescent and young adult participants make independent decisions about learning four categories of conditions (preventable, treatable, adult-onset, and carrier) for themselves utilizing the electronic decision-making tool developed by the study team. Parent participants make their own decisions about which categories of results they want to learn about the adolescent or young adult. Participants can also make granular decisions by excluding or including one or more specific conditions. After making independent choices, the participants complete an additional survey measuring decisional conflict (Table 1. Measures by Study Timepoint).

###### T2 – Joint Discussion and Joint Choices.

A joint decision-making discussion immediately follows T1, during which participants discuss their independent choices. The joint discussion is facilitated by a study team member with clinical genetics or shared decision-making experience. Participants discuss the reasons for their choices, ask questions, and change or confirm their choices as part of the joint decision-making discussion (T2). If, at the end of the joint discussion, an assenting adolescent-parent dyad disagrees on learning certain categories of information, only choices where agreement overlaps are returned. In contrast, if at the end of the joint discussion a consenting adolescent–invited parent dyad disagrees, the consenting adolescent’s choices inform returned results. After making joint decisions, participants independently complete additional survey measures, including a repeat decisional conflict scale (Table 1). Participants are informed that they will receive a survey two weeks later (Decision Change Survey) and will have the option to opt in or out of changing their choices.

At the conclusion of the study visit, adolescents or young adults who decide to learn at least one genomic result provide a saliva or blood sample for DNA collection and sequencing. DNA is extracted from samples by institutional biobank staff and stored for this study’s purposes.

###### T3 – Optional Decision Change Two Weeks After Study Visit.

The third measure of decision stability (T3) occurs two weeks after the study visit when the Decision Change Survey is emailed or sent via text to participants who chose to learn results (Table 1). Adolescent and young adult participants are asked if they want to make any changes to their decisions. Those who choose to keep their original choices or who do not respond within one week to the Decision Change Survey, have their DNA sample shipped to the Broad Institute for sequencing and then to the Laboratory of Molecular Medicine (LMM) for variant analysis based on T2 choices.

Adolescent and young adult participants who want to change their choices at T3, and their parent (if applicable) are reengaged in a joint decision-making conversation where they communicate their new choices, the rationale for the change, ask questions, and change or confirm their new choices. Revised choices are entered into a new joint electronic decision-making tool. Participants’ DNA samples are shipped after completing the new joint decision tool, and the T3 choices are used to ensure that reported results match those choices.

Joint decision-making discussions at T2 and T3 are audio recorded and a subset transcribed for analysis. Topics being explored include whether participants found it helpful to make decisions individually prior to the joint decision-making discussion and whether parents communicate different reasons for wanting to learn “some” results for assenting adolescents vs. young adults.

All sample collection, sequencing, analysis, and result reporting maintain CLIA chain of custody requirements. All results are clinical-grade results, returnable to participants, their providers, their electronic health records, or any combination of the three. The final choices made by each dyad/consenting adolescent are used by LMM to customize reports as needed. Results are returned first to study staff and then to participants, as outlined in Return of Results.

##### SA1 Quantitative Measures: Choices by Assenting Adolescents vs. Consenting Young Adults

The primary outcomes for SA1 are examining adolescent categorical choices on the decision tool (T1), decisional conflict (T2), and decision stability (T3) (whether adolescents’ or young adults’ choices stay consistent or change across the three time points). Prior to making choices about which genetic testing results they would like to learn, adolescent and parent participants complete a series of baseline measures, including demographics, health status, family health history, anxiety, future orientation, everyday discrimination, control preferences, risk perceptions, and study related knowledge questions (Table 1). The adolescent participant also completes a survey that assesses their relationship with their parent. Baseline measures not specific to the primary aims are described in more detail in Supplement 2.

To examine decisional conflict, the Decisional Conflict Scale (DCS) is administered to both adolescents, young adults, and parents at timepoint 1 (T1) and measures personal perceptions of uncertainty in choosing options and modifiable factors contributing to uncertainty, such as feeling uninformed or unsupported (26). The DCS has been modified in this study to examine personal perceptions of uncertainty when participants make decisions about which conditions to learn (Table 1).

At timepoint 2 (T2), following the joint discussion, participants are again asked to complete the DCS, Control Preferences Scale, and study related knowledge questions (see Supplement 2 for description of measures) (Table 1).

At timepoint 3 (T3), adolescent participants are given the one-question (yes/no) Decision Change Survey to change their decisions. The DCS is also administered at T3 if the adolescent changes their decision. Decision stability is measured by whether choices change at T2 or T3. Table 1 lays out the questionnaires and time points for the tools used during this study (Table 1).

A subset of all adolescents and young adults (13–21 years) who indicate the full range of preferences for return of genomic research results available (all results, some results, no results) are invited to participate in a qualitative interview with a member of the study team to explore decision-making, decisional stability, and/or the choice on whether to involve a parent by consenting young adults. We are purposely sampling interviewees to maximize variation in participant age (assenting & consenting) and other demographics, choices, and decision stability. Bioethicists have argued that adolescents nearing or recently afforded the autonomy to make independent choices regarding participation in research are the most appropriate population to assess adolescents’ “genomic citizenship” claims (10). Thus, individuals just on either side of the age of majority are well-positioned to elucidate the ethical and social dimensions of genomic knowledge seeking in the temporal and developmental space between childhood and adulthood.

##### SA1 Qualitative Measures: Choices by Assenting Adolescents vs. Consenting Young Adults

###### Decision Stability Interviews.

Decision stability interviews occur after adolescents and young adults are given the opportunity to change or maintain their choices at T3. Topics include factors that informed adolescents’ and young adults’ decisional preferences regarding return of genomic testing results, reasons for maintaining or changing their choices, confidence in initial decisions and any change in decisions, perceived potential impacts of maintaining or changing choices, and other personal factors that may impact the stability of their decisions.

###### Parental Involvement Interviews.

Consenting participants who either choose to involve a parent in their study visit or participate alone are purposively selected after T3 based on variability in sociodemographic factors to participate in an interview. Topics include the decisional involvement adolescents’ and young adults’ feel parents ought to have when enrolling in genomic research and reasons they did or did not want to engage their parents in their current decision process.

#### Specific Aim 2 – Responses to Genetic Testing Results

Specific Aim 2 (SA2): To examine participants’ psychosocial and behavioral responses to receiving genomic research results that reflect their choices. After genomic sequencing of adolescent participants’ samples collected in SA1, pathogenic/likely pathogenic (P/LP) and negative results that correspond to participants’ final choices from T3 are returned at timepoint 4 (T4) (Table 1). A positive result is defined as having a pathogenic/likely pathogenic variant in a gene that causes or increases the risk of an autosomal dominant or X linked disease or as being heterozygous for a pathogenic/likely pathogenic variant in a pair of genes associated with autosomal recessive disease (carrier result).

##### Return of Results

Adolescents and young adults who receive negative results are sent copies of their reports via secure email or the US postal service. A cover letter is addressed to the parent of assenting adolescent participants, or directly to consenting adolescent participants. We include a written explanation about what negative results mean, the limitations of negative results, and links to online resources that include audio or video information about negative results. Participants are also given the option to contact the study genetic counselor if they have questions about their results.

Adolescents and young adults who have one or more positive result(s) are invited to participate in a scheduled telephone or video disclosure session with their parent (optional for young adults 18–21 years) and a genetic counselor or advanced practice nurse in genetics. After counseling, a paper copy of the laboratory report which meets CLIA/CAP standards, a summary of the counseling session with relevant referral contact information, and links to useful online resources are mailed to the participants.

During the informed consent process, participants are informed that positive results will be placed in participants’ CCHMC electronic health record (as a CLIA-compliant PDF report) and shared with the adolescent’s primary care provider (PCP). Participants may opt in or out of sending negative results to the adolescent’s PCP. Those who opt in designate the health care professional to whom a copy of the laboratory report and cover letter should be mailed.

##### Procedures & Data Collection

###### T4 – Return of Results (RoR) and 1 Week Post RoR.

Following return of results, all adolescents and young adults receiving positive or negative results and their enrolled parents are contacted to complete follow-up surveys one week after result disclosure/anticipated receipt of mailed results to assess psychosocial responses to receiving results at timepoint 4 (T4).

###### T5 – One-year post-RoR for Participants Receiving Positive Results.

Those receiving positive results are invited to participate in a qualitative interview at least three months after receiving their results and complete surveys twelve months after receiving results (timepoint 5 (T5)) to assess behavioral responses, including healthcare utilization, sharing of results, and cascade testing. We will also interview participants who indicated in surveys that they thought they would receive at least one positive result but received only negative results to get a better understanding of reactions to receiving negative results. From our previous research (27), we are expecting approximately 10% of participants to receive positive results, and thus be surveyed at T5. Figure 1 provides a flowchart of the study process.

##### SA2 Quantitative Measures: Response to Results

Quantitative psychosocial and behavioral survey measures collected at one week after return of results (T4) include the Decision Regret Scale and a repeat measure of State Trait Anxiety Inventory (STAI). The STAI measures anxiety as both a current “state” and as a longer term, personal “trait”; we are using the “state” questions throughout this study (28). Decision regret measures distress or remorse after a healthcare decision (29). Risk perceptions are asked again at T4 and match those at T1 except for any questions regarding perceived likelihood of receiving negative or positive results, as return of result has already occurred by T4.

Four additional questionnaires developed or adapted by the study team, including Perceived Utility, Results Congruency, Sharing of Results, and Family Testing (30), are also administered at T4. Perceived Utility measures participants’ perceived usefulness of the return of result information and return of result process, while Results Congruency measures whether participants feel the results received match what they were expecting. Sharing of Results and Family Testing assess behavioral responses after return of results, including sharing of results with at-risk family members, cascade testing, and follow-up medical appointments related to the result received.

Follow-up surveys at 12 months (T5) for participants receiving positive results will include repeat measures of sharing of results and family cascade testing.

##### SA2 Qualitative Measures: Response to Results

Qualitative interviews at least three months after return of positive results focus on an in-depth exploration of participants’ reactions to, perceived utility, risks, benefits, and implications of learning results. Interviews are audio recorded and transcribed. We invite all participants who receive positive results to participate in an interview, as the type of result, age, sex, and recruitment pathway may vary considerably. We ask participants how they might answer each portion of the decision tool now that they have received positive results. This information will contribute to our understanding of decision stability, the reliability of the electronic decision-making tool, and the impact of specific types of results on decisional preferences. We also ask whether desired parent or adolescent involvement in the decision-making process has changed after receiving results, and if so, how. Finally, we ask participants about intended and actual behavioral responses to genomic test results, including sharing of results, medical appointments related to returned test results, and future reproductive or life plans.

##### Quantitative Data Analysis

###### SA1 – Choices by Assenting Adolescents vs. Consenting Young Adults

Data analysis for the quantitative portion of SA1 will explore categorical choices about learning genomic results, decisional conflict, and decision stability.

Choices will be operationalized for analysis purposes as the decision to receive all results, some results, or no results. If numbers permit, we will further stratify by categories of choices (preventable, treatable, adult-onset, and carrier). Decisional conflict is a continuous measure and will be assessed at T1, T2, and T3 (if the adolescent changes their decision at T3). Decision stability is defined as the consistency of choices (regardless of choices made) at T1, T2, and T3. We will explore all outcomes (choices, decisional conflict, and decision stability) between assenting adolescents and consenting young adults.

To examine the relationship among choices, decisional conflict, and decisional stability, we will use logistic regression and include covariates as appropriate with choices (all or some) as the outcome variable and decisional conflict scores at T1 and decision stability as independent predictors. Similar analyses will be performed with decision stability as the outcome. These analyses will be performed separately within assenting adolescents and consenting young adults, as well as within parents.

Additionally, we will compare the proportion of adolescents and young adults who do and do not have decisional conflict scale (DCS) scores higher than 25 at T1 and T2, as well as whether DCS scores differ between adolescents and young adults. Initial analyses will use contingency tables. If covariates are associated with DCS scores, then we will use logistic regression. We will also examine whether overall DCS scores change for all participants between T1 and T2.

###### Sample Size

We plan to enroll a total sample of 240 assenting adolescent/parent dyads and 200 consenting adolescents and young adults from all recruitment avenues to maximize our ability to achieve near equal numbers of participants representing each year of age from 13–21. While primary quantitative analyses for SA1 would be sufficiently powered with smaller sample sizes, these numbers will permit us to more closely examine age effects and account for covariates in the analyses. For all sample size calculations, alpha = 0.05.

We will have 80% power to detect a difference as small as 10.3 in the Decisional Conflict Scale score between those choosing all vs. some genetic testing results and a difference as small as 4.5 in the DCS score between assenting adolescents vs. consenting young adults. Both effect sizes are calculated based on t-tests with 25% increase in effect size to account for non-parametric tests.

In our previous research, we found that 20% of adolescents changed their decisions during the joint decision-making discussion (17, 18). Assuming a similar rate of decisional instability, we will have 80% power to detect an odds ratio as small as 2.4, if those who choose to learn all genetic testing results have 10% instability. This difference is reasonable, as the rate of decisional instability between independent and joint decision making in eMERGE III was 2% and 55% among those independently choosing all vs. some genetic testing results.

##### SA2 – Response to Results

Data analysis for the quantitative portion of SA2 will initially explore psychosocial and behavioral responses to receiving chosen genomic research results for adolescents and young adults. We will test whether there are relationships between result type and scores on the State Trait Anxiety Inventory (STAI) and Decision Regret Scale surveys. STAI is also measured at baseline T1 timepoint, and we will explore variations in survey scores over time and by result received. We will also examine behavioral actions in surveys of Sharing of Results and Family Testing by result received. Finally, we will examine Perceived Utility, Results Congruency, and changes in Risk Perceptions or Study Knowledge questions after receiving results. Risk Perceptions and Study Knowledge questions are measured at multiple timepoints, and we will explore variations in scores over time and by result received. We will test whether covariates (e.g., age, sex, race, income, education, etc.) are associated with psychosocial and behavioral responses and type of result returned.

Analysis will continue with examination of decision regret after return of results and its relationship to decisional instability by type of participant choice. Decisional conflict scores were skewed in our preliminary findings from the eMERGE III study (19), and we anticipate that decision regret will also be skewed in the current study. Thus, we will use the nonparametric Wilcoxon rank sum test to test whether decision regret is associated with decision instability. Analyses will be performed separately for those receiving positive and negative results as well as by assenting adolescents and consenting young adults.

We will also test whether covariates (e.g., adolescent age when results are returned, sex, race, income, knowledge, and change in state anxiety from baseline) are associated with decision regret. If covariates are associated with regret, we will use generalized estimating equations (GEE) so that the appropriate data distribution may be specified, and covariates included. To test whether a positive result modifies the relationship between regret and instability, we will use GEE with an interaction term to evaluate whether the relationship is modified.

##### Sample Size

To estimate power for analysis of SA2, we dichotomized decisional regret into those who had a score above and below 10, based on skewed decision regret seen in eMERGE III. Given a sample size of 200 consenting young adults, we will have 80% power to detect an odds ratio as low as 3.3 if the rate of instability among those without regret is 8% at alpha = 0.05. Based on our preliminary data, these effects are reasonable.

##### Qualitative Data Analysis

###### SA1 - Choices by Assenting Adolescents vs. Consenting Young Adults & SA2 - Response to Results

Interview transcripts and joint decision-making transcripts will be predominantly analyzed using the general principles of grounded theory (31, 32). Grounded theory approaches seek to inductively uncover social processes and conditions that underlie phenomena and unravel their consequences. This method is especially suited to studies where the general phenomenon and its processes are emerging, and in which constant comparisons between data from multiple samples and sources are a critical part of the research agenda (33). This inductive analytic method is also effective for revealing embedded normative assumptions and values implicit in participants’ worldviews (34, 35).

Once transcribed, we will begin inductive open coding of segments of text with a handful of transcripts. Specific codes will be derived using both deductive strategies (built on existing scholarship and then applied to the transcripts) and inductive strategies (built directly from the transcript text).

A coding manual will be developed with definitions and illustrations of each code. These coded transcripts will be carefully reviewed to clarify the meaning of particular codes and create clear decision-making rules related to coding processes. A constant comparison approach will be used to code the transcripts. The strategy of making constant comparison of codes and themes within and between sets of interviews is necessary for generating sensitive analyses and nuanced theories of how participants understand the value of genomic information for themselves and their families (32).

Analysis of qualitative interviews will utilize these same procedures and methods for both SA1 and SA2.

## Discussion

We are conducting a mixed-methods longitudinal clinical trial to explore adolescent, young adult, and parent joint decision-making when participants aged 13–21 are given the opportunity to learn genetic research results about themselves. We will investigate best practices for engaging adolescents and young adults in decisions to learn genomic research results, as opposed to relying solely on parents to make decisions for the adolescents. Additionally, we will investigate the impact on adolescents and young adults of learning genomic research results. Results from this study will also help inform the process of returning genomic research results on a large-scale basis to a range of populations recruited from a variety of settings.

While previous studies have begun or are poised to return genomic research results to pediatric populations (27, 36, 37), we believe this current study has unique factors that will contribute to supporting best practices and ethical considerations when engaging adolescents and young adults in genomic testing decisions. One of the strengths of our study is the electronic decision-making tool and its associated educational videos, which feature options for adolescents, young adults, and parents to make informed, customizable choices about learning genetic research results for the adolescent that are aligned with their values. With the tool being readily accessible online, it has the potential to be scaled up for use in decision-making scenarios in other settings.

Additionally, we are actively engaging local primary care clinic patients and community research advocates to generate interest and refer community who identify as members of racial minority groups that have been historically underrepresented in genomic research (Halbert & Harrison, 2018; Saulsberry & Terry, 2013; Zhu et al., 2020). Research conducted with these groups may be more critical to the moral imperative for bidirectional development and translation of participant-researcher relationships (Blumling et al., 2021). Promoting informed decision-making among young research participants is one of the benefits of the current study methods. Study participants are heavily involved in the decision-making process, which offers a unique method for choosing which research results they desire to learn about themselves; thus, promoting their independence and emerging autonomy in research. While only results that overlap between an assenting adolescent and their parent are returned due to legal constraints, we conduct a facilitated discussion between adolescent-parent dyads to encourage sharing of thoughts, desires, and questions related to genetic research result choices. This enables us to ensure that the adolescent voice is noted and considered by the parent prior to making any final choices that may impact the adolescent’s health going forward.

Finally, this study offers a unique opportunity for participants to select their preference for two methods of study visit and sample collection. Participants have the opportunity to select either an in-person or virtual study visit, which is especially helpful for individuals who may live farther from the study sites or have transportation barriers. Additionally, participants may contribute their perspectives while opting out of providing a DNA sample and they can select whether they want to provide a blood or a saliva sample for testing, which may alleviate stressors for some participants associated with a standard blood draw. This flexibility will help with recruitment and perhaps help with engagement from communities with historic mistrust of medical research. We believe that the information learned from utilization of our electronic decision-making tool, community engagement, and emphasis on joint decision-making will fill a significant gap in current knowledge. Additionally, we expect to make a significant contribution to the ethical and policy debates about predictive pediatric genetic testing, and in particular, adolescents’ and young adults’ preferences and involvement in the return of genomic sequencing results, particularly for adult-onset disorders and carrier status.

## Limitations

We are actively recruiting and enrolling participants who identify as members of racial minority populations and are striving to ensure that no more than 65% of our participants self-identify as white. However, participants may not be representative of the general population. Those who choose to participate may be more likely to want to learn results than those who do not participate. Additionally, this study is being performed in a single metropolitan region in the Midwest and participation is limited to those with English proficiency. Future studies with a larger sample size from a broader geographic range will be necessary to validate findings from our current study.

## Conclusion

The Engaging Adolescents in Decisions About Genomic Results study offers adolescents, young adults, and their parents the opportunity to make decisions about learning genetic testing results about the adolescent/young adult. Our study will add a unique contribution to the small body of literature on pediatric and emerging adult decision making regarding learning personal genomic information. We will expand prior literature primarily focused on genetic testing in healthy at-risk adolescents with a known family history of a single gene disorder. Filling a glaring gap in the literature, we will be able to address differences before, during and after the magical age of 18 when making decisions about genomic screening and learning results for adult-onset disease risk and carrier status.

## Supplementary Material

Supplement 1

## Figures and Tables

**Table 1:** Measures by Study Timepoint

Data Collection Method	Domain	Measures	Time Points (T1–5)					Completed By
	**SA1: choices about learning genomic results, decision stability, and decisional conflict between assenting adolescents and consenting young adults**							
Quantitative Survey	Demographics	Developed Measure: gender, sex, age, race, ethnicity, education level, family member in healthcare, number of children, household members, health status, resources, trust in healthcare and research, and genetics experience	T1					Adolescent/Young Adult (A/YA), Parent (P)
	Generalized Anxiety	Validated Measure: Generalized Anxiety Disorder Scale 7-item (GAD-7)	T1					A/YA, P
	Discrimination	Validated Measure: Everyday Discrimination Scale	T1					A/YA, P
	Family History	Developed Measure: Family history, including relationships, health, and ancestry	T1					A/YA, P
	Future Orientation & Delay Discounting	Validated Measure: Future Orientation Scale	T1					A/YA
	Study Knowledge Questions	Developed Measure: Questions related to understanding of genetics	T1	T2		T4		A/YA, P
	State/Trait Anxiety	Validated Measure: State-Trait Anxiety Inventory (STAI) 6-item Short Form	T1			T4		A/YA, P
	Control Preferences	Developed Measure: Questions related to control preferences when learning genomic information	T1	T2				A/YA, P
	Risk Perception	Developed Measure: Questions related to risk of developing categories of diseases	T1			T4		A/YA, P
	Parent Trust, Communication, and Alienation	Validated Measure: The Inventory of Parent and Peer Attachment	T1					A/YA
	Decision Change	Decision Change Option - Yes/No Question: At two weeks post original decision (T2), adolescents and young adults are given an option to change their original choices by selecting Yes or No in a one-question survey			T3			A/YA
	Decisional Conflict	Validated Measure: Decisional Conflict Scale (DCS)	T1	T2	*T3			A/YA, P
Qualitative Interview	Decisional Stability	Questions related to decision-making process and changes at T2 and T3			Between T3 – T4			Subset of A/YA
	Parental Involvement	Questions related to consenting adolescent decision to invite or not invite a parent			Between T3 – T4			Subset of A/YA
	**SA2: psychosocial and behavioral responses to receiving genomic research results**							
Quantitative Survey	Decision Regret	Validated Measure: Decision Regret Scale				T4		A/YA, P
	Results Congruency	Developed Measure: Questions related to result expectations				T4		A/YA, P
	Perceived Utility	Developed Measure: Questions related to satisfaction & utility of RoR				T4		A/YA, P
	Sharing of Results	Developed Measure: Questions related to reasoning of sharing or not sharing results				T4	***T5	
	Family Testing**	Developed Measure: Questions related to cascade testing in family				T4	***T5	
Qualitative Interview	Response to Positive Results	Questions related to impact of P/LP or carrier results disclosure on adolescent, parent, and extended family				T4		Subset of A/YA

A = Adolescent; P = Parent (required for assenting adolescent; optional for consenting adolescent)

* DCS only measured if participant selects Yes to change their choices to learn genomic testing results in Decision Change Option

** This survey is only sent to individuals with a P/LP or carrier result

*** T5 timepoint only occurs with individuals with a P/LP or carrier result
