# Supplementary material for: Engaging Adolescents and Young Adults in Decisions About Return of Genomic Research Results: a mixed-methods longitudinal clinical trial protocol
Source: Res Sq. 2023 Apr 27:rs.3.rs-2819191. Preprint. [Version 1] doi: 10.21203/rs.3.rs-2819191/v1 (PMC10168476; doi:10.21203/rs.3.rs-2819191/v1)
Supplement: Supplement 1 [file NIHPPrs2819191v1-supplement-1.pdf]

Supplementary Files

This is a list of supplementary files associated with this preprint. Click to download.

- ProtocolSupp1.docx
- ProtocolSupp2.docx
